# Paired-Associative Stimulation-Induced Long-term Potentiation-Like Motor Cortex Plasticity in Healthy Adolescents

**DOI:** 10.3389/fpsyt.2017.00095

**Published:** 2017-05-29

**Authors:** Jonathan C. Lee, Paul E. Croarkin, Stephanie H. Ameis, Yinming Sun, Daniel M. Blumberger, Tarek K. Rajji, Zafiris J. Daskalakis

**Affiliations:** ^1^Temerty Centre, Centre for Addiction and Mental Health, Toronto, ON, Canada; ^2^Mayo Clinic Depression Center, Department of Psychiatry and Psychology, Mayo Clinic, Rochester, MN, United States; ^3^Hospital for Sick Children, Toronto, ON, Canada

**Keywords:** developmental neuroplasticity, adolescent psychiatry, paired-associative stimulation, motor cortical plasticity, motor-evoked potentials, cortical silent period

## Abstract

**Objective:**

The objective of this study was to evaluate the feasibility of using paired-associative stimulation (PAS) to study excitatory and inhibitory plasticity in adolescents while examining variables that may moderate plasticity (such as sex and environment).

**Methods:**

We recruited 34 healthy adolescents (aged 13–19, 13 males, 21 females). To evaluate excitatory plasticity, we compared mean motor-evoked potentials (MEPs) elicited by single-pulse transcranial magnetic stimulation (TMS) before and after PAS at 0, 15, and 30 min. To evaluate inhibitory plasticity, we evaluated the cortical silent period (CSP) elicited by single-pulse TMS in the contracted hand before and after PAS at 0, 15, and 30 min.

**Results:**

All participants completed PAS procedures. No adverse events occurred. PAS was well tolerated. PAS-induced significant increases in the ratio of post-PAS MEP to pre-PAS MEP amplitudes (*p* < 0.01) at all post-PAS intervals. Neither socioeconomic status nor sex was associated with post-PAS MEP changes. PAS induced significant CSP lengthening in males but not females.

**Conclusion:**

PAS is a feasible, safe, and well-tolerated index of adolescent motor cortical plasticity. Gender may influence PAS-induced changes in cortical inhibition. PAS is safe and well tolerated by healthy adolescents and may be a novel tool with which to study adolescent neuroplasticity.

## Introduction

Adolescence is a period of tremendous neuronal plasticity characterized by synaptic pruning, and axonal myelination as the brain matures into its adult state (1, 2). Therefore, adolescence may be a unique period when vulnerability to disease and responsiveness to intervention are both enhanced (3, 4). Consequently, improving our understanding of normal neuronal processes in adolescence could enable researchers and clinicians to both identify abnormal development and develop therapeutic interventions with which to correct it.

Psychiatric disorders such as depression and schizophrenia typically arise in late adolescence (5–8) and are associated with disrupted long-term potentiation (LTP) (9–11). LTP is a form of neuroplasticity that occurs when co-firing neurons become increasingly associated over time (12). Although few paradigms permit direct assessment of LTP in humans, paired-associative stimulation (PAS) is one such method (13). Player et al. (11), for instance, showed that PAS did not produce focal motor LTP in depressed adult patients, an effect which was ameliorated with treatment (14). Similarly, Batsikadze et al. (15) showed that serotonin modulates PAS-induced spike-dependent plasticity, enhancing PAS-induced motor facilitation. These findings highlight the potential utility of PAS as a neurophysiological technique to detect impaired plasticity.

In the original PAS experiment, Stefan et al. (13) administered single-pulse transcranial magnetic stimulation (TMS) over the left motor cortex in the area corresponding to the right abductor pollicis brevis (APB), taking an average of motor-evoked potentials (MEPs) to establish baseline motor cortical activity. Peripheral nerve stimulation (PNS) was then delivered to the median nerve before TMS, at an interstimulus interval (ISI) of 25 ms. An ISI of 25 ms is estimated to allow for the synchronous arrival of both inputs to the motor cortex and was shown to induce lasting motor cortical plasticity (13).

Following PAS, participants receive single-pulse TMS again over the left motor cortex and MEPs recorded from the contralateral APB. The average post-PAS MEPs are computed and a ratio of post-PAS to pre-PAS MEPs calculated. In instances where this ratio exceeds 1, denoting that the post-PAS average exceeded the pre-PAS average, PAS is said to have induced motor facilitation. Although the original PAS paradigm demonstrated PAS in the contralateral APB (13), it should be noted that PAS also influences the ipsilateral hemisphere (16). PAS-induced motor facilitation is a direct measure of LTP *in vivo* in humans (13).

PAS may also induce changes in GABAergic neurotransmission. Although GABA causes membrane depolarization (excitation) early in development, it induces hyperpolarization (inhibition) in the mature brain (17). Pediatric TMS studies suggest that GABA-dependent cortical inhibition is disrupted in neuropsychiatric illnesses such as depression (18, 19). Croarkin et al. (18), for instance, showed that depressed adolescents with pretreatment deficits in long-interval cortical inhibition were less likely to respond to treatment with antidepressants. This developmental change during maturation, and its association with the onset of neuropsychiatric illness, highlights the need to study cortical inhibitory processes across the lifespan.

One measure of GABA_B_ cortical inhibition is cortical silent period (CSP) which PAS lengthens (20). Both Stefan et al. (13) and Sale et al. (20), for instance, showed that PAS lengthened CSP in adults. Whether PAS would lengthen CSP in healthy adolescents remains to be seen, though one meta-analysis of age-related changes in TMS measures suggested that age did not change CSP length (21).

Data from pediatric TMS studies suggest single- and paired-pulse TMS is a safe, tolerable, and minimal risk intervention (22–25). Further work in this area using novel investigational techniques is critical to understand liabilities to psychiatric illness in this developmental stage and enhance our understanding of disease mechanisms (2, 26, 27). Damji et al. (28) demonstrated that PAS was feasible and tolerable in a pediatric population. Their study, however, focused on a younger sample comprised mainly of children (mean age 12 years). Given the tremendous amount of neurocognitive development thought to occur during the teen years, we sought to establish whether PAS was a feasible and tolerable protocol with which to elucidate healthy developmental neuroplasticity in an adolescent sample. In addition, we sought to determine whether PAS would lengthen CSP in adolescents, as in adults.

We also sought to examine factors associated with neuroplasticity including sex, age, and socioeconomic status (SES). For example, previous work has demonstrated sex differences in synaptic connectivity in the frontal cortex in response to chronic (29) and prenatal stress (30). In humans, Tecchio et al. (31) found an age–sex interaction with older post-menopausal females showing no PAS-induced motor potentiation when compared to younger females and males of all ages. SES may also impact neuroplastic processes in the adolescent brain. Adolescents arising in impoverished conditions show lower academic achievement than higher SES peers, suggesting that SES may interact with this behavioral measure of neuroplasticity (32, 33).

Therefore, the aim of this study was twofold: (1) to determine whether PAS was feasible in adolescents and (2) to characterize potential external factors influencing PAS outcomes. To determine the feasibility of PAS in adolescents, we evaluated PAS-induced excitatory (motor facilitation) and inhibitory (CSP) plasticity in healthy adolescents, reporting on these measures as well as participant dropout. We also evaluated whether external factors such as sex, age, SES, and academic achievement influenced PAS-induced plasticity. We hypothesized that PAS would induce motor facilitation in healthy adolescents. Moreover, we hypothesized PAS would lengthen CSP in adolescents, as in adults, in the absence of evidence to the contrary.

## Materials and Methods

This study was approved by the Research Ethics Board of the Centre for Addiction and Mental Health (CAMH) in Toronto. In accordance with the recommendations of the Declaration of Helsinki, all participants provided their written informed consent prior to participation. The protocol was approved by the Research Ethics Board of the CAMH in Toronto, ON, Canada.

To evaluate the feasibility of using PAS in healthy adolescents, we delivered PAS according to the methods described by Stefan et al. (13). All data were collected at the Temerty Centre for Therapeutic Brain Intervention at CAMH. A research associate provided full information about the study objectives, procedures, and known potential adverse events. Participants were invited to describe study procedures and their understanding of the implications for their circumstances to assess capacity. Capable participants aged 16–19 provided their written informed consent before study commencement. Those aged 13–16 gave their written informed assent while a parent or guardian gave written informed consent for their child to participate.

### Participants

We recruited adolescents aged 13–19 from community agencies, schools, and the Internet. Research associates contacted interested respondents who called the Temerty Centre for Therapeutic Brain Intervention, provided a non-standardized introduction of the study objectives, and described the potential risks associated with TMS. Respondents completed a standardized TMS safety screen and had the opportunity to ask questions about the study to which research associates provided non-standardized responses.

Eligible participants were English speakers, had parents who spoke conversational English, were right handed [according to the Oldfield handedness interview (34)], and were capable, or accompanied by a capable parent/guardian, to consent to study participation. Participants were ineligible if they had a known history of seizures, diagnosed psychiatric disorders, substance use in the preceding 3 months, unstable medical or neurological condition, intellectual disability, or were currently pregnant.

### Clinical Assessments

Participants completed an interview with the Mini International Neuropsychiatric Inventory for Children and Adolescents (MINI-KID) 6.0 to rule out psychiatric illness (35). The MINI-KID 6.0 possesses excellent sensitivity and specificity for alcohol abuse and dependence (sensitivity 0.94, specificity 0.96) and drug abuse and dependence (sensitivity 0.98, specificity 0.93), excellent inter-rater (AUC 1.00, *K* 1.00), and test–retest (AUC 0.99, *K* 0.98) reliability (35). We used the MINI-KID 6.0 for all participants. In participants under 16, we obtained parental responses with the same measure.

The Wide Range Achievement Test 4 (WRAT-4) was used to assess academic achievement (36). The WRAT-4 is a structured neuropsychological assessment instrument that examines reading, sentence comprehension, spelling, and math computation. Age- and grade-based norms are available, and percentile rankings are calculable from raw scores (36).

All participants provided their parent’s level of education and occupation. We calculated the Hollingshead Index (37), which measures SES based on a score comprised of ratings of parental occupation and education. Ratings of parental occupations range from a score of 9 (for proprietors of large businesses and professionals) to 1 (for menial service workers and laborers). Ratings of parental education range from 7 (graduate degree) to 1 (less than seventh grade). Two research associates assigned ratings independently based on categories described in Hollingshead’s original paper. We resolved discrepant ratings by consensus. We calculated the Hollingshead Index by multiplying the occupation score by a factor of 5 and the education score by a factor of 3. In two-parent homes, parental scores are summed and divided by 2 yielding a minimum possible score of 8, and a maximum possible score of 66.

### Electromyography (EMG)

Each neurophysiology testing session lasted 1.5 h. Participants sat in a comfortable chair, with a cushion in their lap, and right forearm exposed. We prepped the thumb and volar surface of the forearm with an alcohol wipe for recording. We collected EMG data from disposable disks in a tendon-belly arrangement. Participants were directed to relax their right hand for the entirety of the study. EMG recording and speakers at high gain served as another measure of muscle activity throughout testing. The EMG signal was amplified (Intronix Technologies Corporation Model 2024F, Bolton, ON, Canada), filtered (band pass 2 Hz–2.5 kHz), digitized at 5 kHz (Micro 1401, Cambridge Electronics Design, Cambridge, UK) and stored in a laboratory computer for offline analysis.

### Transcranial Magnetic Stimulation

We delivered single-pulse TMS to the left motor cortex with a 7-cm figure-of-eight coil and a Magstim 200 stimulator (Magstim Company, Whitland, UK). We used a moderately suprathreshold stimulus to identify the optimal APB stimulation location, marking it with a felt-tipped marker to ensure consistent coil placement across trials. The handle of the coil pointed backward at 45° to the mid-sagittal line and perpendicular to the central sulcus.

We determined the resting motor threshold (RMT) at the optimal stimulation position. We defined the RMT as the minimum stimulus intensity needed to produce a response of at least 50 µV in the relaxed APB in 5 of 10 consecutive trials. The stimulus intensity required to evoke a 1-mV peak-to-peak response (SI_mV_) was then determined. Stefan and colleagues found the SI_1mV_ to be approximately 120% of the RMT (13). We defined the SI_1mV_ as the stimulus intensity required to evoke, on average, a MEP of 1 mV in amplitude over 15 trials. If the average MEP over 15 trials was not 1 mV upon using 120% of RMT, the intensity was adjusted in 2% increments until we determined the SI_1mV_. Upon determining the SI_1mV_ to be used in the study, we measured MEP over 20 trials and computed the average MEP.

### Median Nerve Stimulation

The median nerve provides sensory innervation to the thumb, index, long, and medial edge of the fourth finger, and motor innervation of the thenar and two lumbrical muscles. We delivered constant current square wave pulses by a standard, cathode proximal stimulation block. To determine the sensory threshold, we asked participants to close their eyes and respond affirmatively each time they noted a sensation. We defined the sensory threshold as the lowest stimulus intensity evoking a positive response. We set the pulse width to 200 µs and the stimulus intensity at 300% of the sensory threshold. As attention modulates PAS-induced plasticity, we employed the method described by Stefan et al. (38), asking participants to count the total number of median nerve stimuli they had received during the study process and at the end of the study.

### Experimental Design

**Figure 1:**
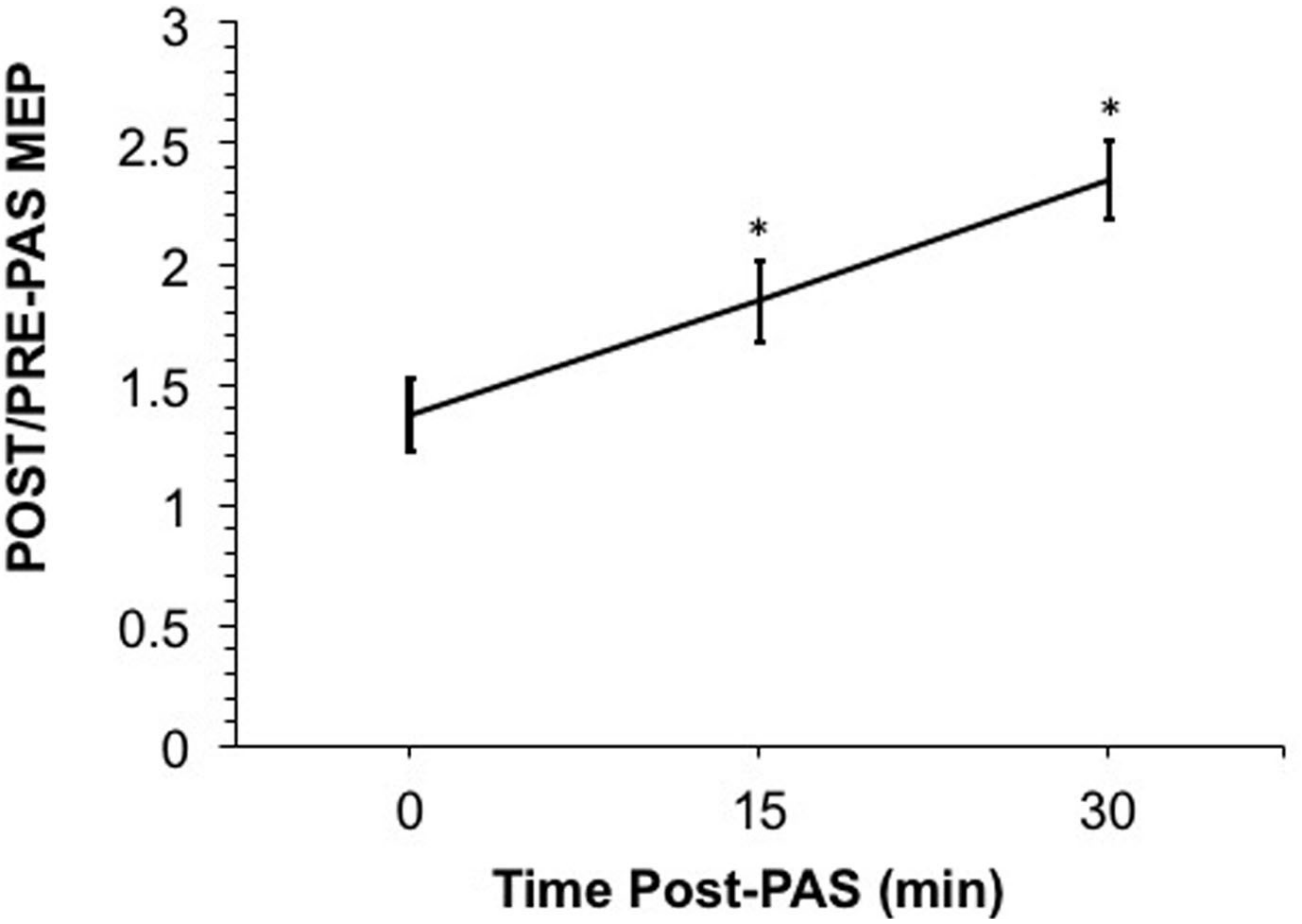
**Shows the ratio of the raw post-paired-associative stimulation (PAS) motor-evoked potential (MEP) over the raw pre-PAS MEP**. A ratio over 1 suggests a post-PAS increase in MEP. PAS induced significant motor facilitation at 15- and 30-min post-PAS (*p* = 0.013 and *p* = 0.007, respectively); however, MEPs at 0-min post-PAS were not significantly different from baseline once log-transformed. Error bars depict SEM.

Participants underwent RMT, and SI_1mV_, and CSP testing at baseline. PAS consisted of median nerve stimulation at 300% sensory threshold followed by TMS at the SI_1mV_ intensity at an ISI of 25 ms. Stefan et al. (13) previously showed that an ISI of 25 ms can induce motor facilitation. This interval is thought to represent the time required for a median nerve stimulus to reach the motor cortex contemporaneously with the TMS pulse. In the present study, we delivered 180 pairs of stimuli at 0.1 Hz. We calculated the mean of 20 MEPs evoked by the SI_1mV_ at 0, 15, and 30 min after PAS. Figure 1 showed the ratios of mean MEPs to baseline mean MEP. We measured CSP at baseline and 30 min post-PAS. Participants were asked to grasp a pinch gage at 20% of their maximal grip strength while we delivered single-pulse TMS at 140% RMT intensity at a frequency of 0.1 Hz. Participants completed 10 trials for each CSP session.

### Data Analysis

We calculated descriptive statistics of sample demographics and WRAT-4 scores. We used a non-parametric ANOVA test (the Friedman test) to compare mean MEPs evoked at baseline, 0-, 15-, and 30-min post-PAS since ratio data were not normally distributed. We then completed *post hoc* paired-comparisons between MEP values with a two-tailed Wilcoxon signed rank test between each post-PAS time point and baseline, correcting for multiple comparisons (i.e., three) with an *a priori* significance level of 0.0167. To evaluate PAS-induced changes in CSP, we used repeated measures ANOVA with “PAS session” as a within-subject factor and “sex” as a between-subject factor. We chose parametric testing for this measure since, unlike ratio data, CSP duration was normally distributed. The CSP duration was defined as the time between onset of MEP and return of voluntary contraction on EMG and determined by visual inspection as previously described (39). For CSP, we set the *a priori* significance level to 0.05. We completed all analyses with SPSS 22 (IBM Corp., Armonk, NY, USA, 2013).

To evaluate the impact of various independent variables on MEP potentiation, we evaluated Spearman’s correlation between the average PAS ratio and ordinal independent variables [SES (Hollingshead Index), WRAT scores, age, and sex]. We used the Wilcoxon rank-sum test to compare group differences for MEP potentiation due to categorical variables, which include gender and Hollingshead high vs. low using a median split [low ≤54 (*n* = 17), high >54 (=17)].

## Results

Thirty-six eligible respondents contacted our lab for potential participation. One respondent aged out of our age range shortly after her telephone interview and became ineligible. One participant declined to undergo PAS due to a high RMT that would have necessitated a greater than maximal stimulator output intensity for PAS. He did not endorse side effects from TMS before or after termination.

Thirty-four eligible participants completed the study. Participants were aged 13–19 years (mean = 17.7 ± 1.3). Twenty-one (61.8%) participants were female. Males and females did not differ in age (*p* = 0.45). Table 1 displays demographic information, ethnicity, WRAT-4 scores, stimulation parameters, and mean pre-PAS MEP. All participants were healthy and demonstrated average or above average academic achievement based on the MINI-KID and WRAT-4. Scores on the Hollingshead Index ranged from 24 to 66 (mean 51.0 ± 10.6). Participants guessed that they had received a mean of 177 ± 19 peripheral nerve pulses. A one-sample *t*-test did not reveal a significant difference between participant guesses and the actual total number of 180 pulses (*t* = −0.907; df = 33; *p* = 0.371).

**Table 1:** **Participant characteristics and neurophysiology (*N* = 34)**.

Age (years ± SD)	17.7 ± 1.3
	*N* (%)
Age 13–16	7 (20.5)
Age 17–19	27 (79.4)
Gender	
Female	21 (61.7)
Ethnicity	
Caucasian	6 (17.6)
East Asian	17 (50.0)
South Asian	3 (8.8)
African/Afro-Caribbean	7 (20.6)
Latino	1 (2.9)
Mini International Neuropsychiatric Inventory for Children and Adolescents 6.0 Diagnoses	0 (0%)
Wide Range Achievement Test 4 standard scores (mean score ± SD)	
Word reading	110.1 ± 16.1
Sentence completion	97.1 ± 12.5
Spelling	115.0 ± 17.9
Math computation	108.2 ± 18.4
Mean Hollingshead Index	50.6 ± 11.5
Mean baseline motor-evoked potential	0.82 ± 0.25 mV
Mean resting motor threshold	57.4 ± 10.7% stimulator output
Mean SI_1mV_	71.4 ± 11.9% stimulator output
Mean sensory threshold	1.1 ± 0.9 mA
Mean peripheral nerve stimulation intensity	3.4 ± 2.7 mA
Mean stimulation count	177 ± 19

### Stimulus Intensity, RMT, and Sensory Threshold

Mean RMT was 57.4 ± 10.7% of stimulator output, mean SI_1mV_ was 71.4 ± 11.9%, the mean sensory threshold was 1.1 ± 0.9 mA, and the mean PNS intensity was 3.4 ± 2.7 mA. We did not find any significant association among age, sex, Hollingshead and WRAT scores with mean RMT, mean SI_1mV_, mean sensory threshold, or PNS intensity.

### PAS-Induced MEP Potentiation and CSP Changes

We confirmed our hypothesis that PAS would induce LTP-like plasticity in healthy adolescents. The ratio of post-PAS/pre-PAS MEPs exceeded one as shown in Figure 1. The results of our Friedman test showed that there was a significant main effect of time (χ^2^ = 13.17, df = 3, *p* < 0.005). Moreover, *post hoc* testing with a two-tailed Wilcoxon signed rank test revealed significant differences in MEPs from baseline at 15- and 30-min post-PAS (with exact *p* values of *p* = 0.0020 and *p* = 0.0017, respectively). These differences were significant even after Bonferroni correction for multiple comparisons with a threshold of *p* = 0.0167. MEPs were not significantly different from baseline at 0-min post-PAS. No correlation or group comparison of MEP potentiation with Hollingshead scores, WRAT scores, sex, and age showed any significant effect.

**Figure 2:**
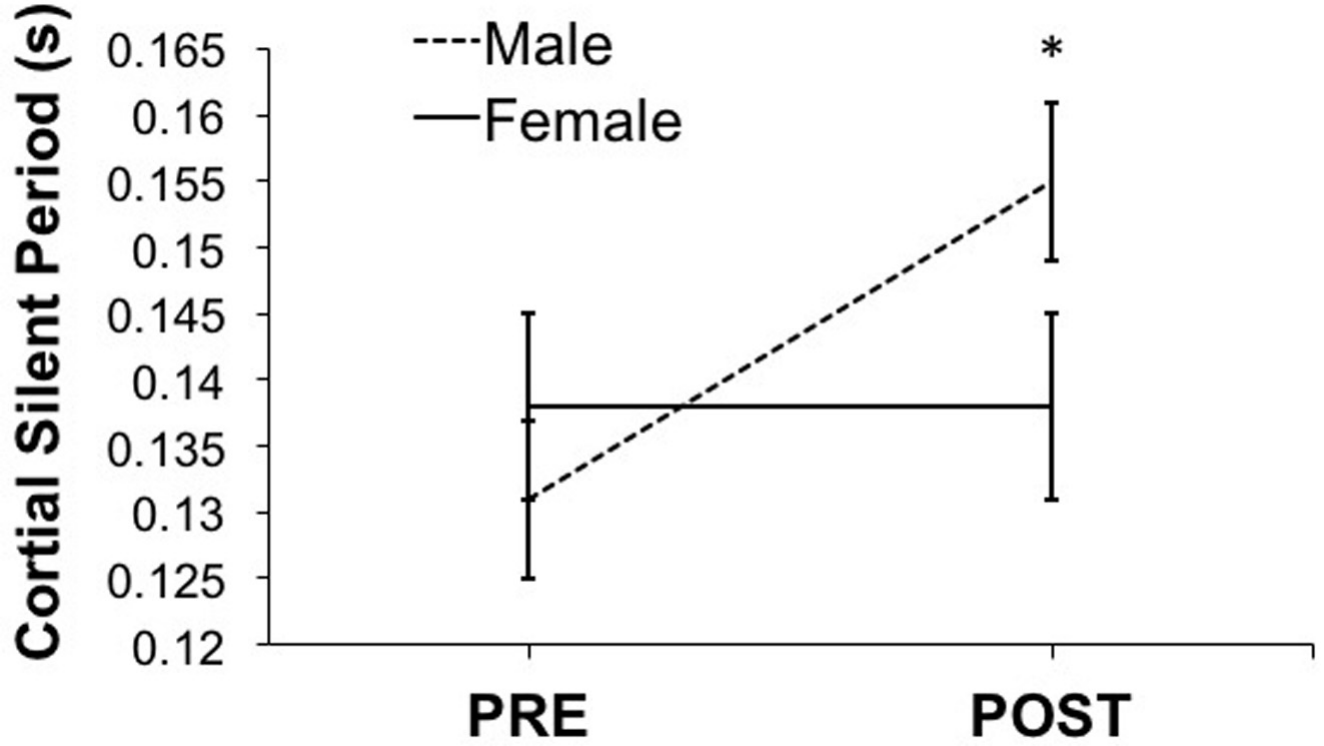
**Shows the effect of paired-associative stimulation (PAS) on cortical silent period (CSP)**. Male, but not female, adolescents showed significant post-PAS CSP lengthening (male > female, *p* < 0.05). Error bars depict SEM.

As shown in Figure 2, PAS caused a significant CSP change (*F* = 7.67, *p* = 0.009). We found a significant interaction between PAS session (PRE and POST) and sex (*F* = 7.16, *p* = 0.011). Males showed significant CSP lengthening post-PAS (mean difference = 0.024, *p* = 0.014). Conversely, females showed no PAS-induced CSP changes (mean difference = 0.0004, *p* = 0.929). CSP was uncorrelated with age, Hollingshead score, or WRAT score.

## Discussion

To our knowledge, this is one of the first studies demonstrating that PAS is both feasible and tolerable in an adolescent sample (28, 40). Damji et al. (28) showed that PAS was safe and tolerable, and produced motor facilitation in a majority of their study participants. Their sample consisted of 28 participants [20 males (71.4%)] with a mean age of 12 years. By contrast, our sample consisted of 34 participants, a majority of whom were female [*n* = 21 (61.8%)] with a mean age of 17 years. Our mean sample age was, therefore, situated in a continuum between Damji et al. (28) and Stefan et al. (13). While Damji et al. (28) delivered 90 pairs of stimuli during PAS, we administered 180 pairs in an adolescent sample. We also evaluated the effect of PAS on CSP, a measure of GABA_B_-mediated inhibitory tone. Finally, we examined whether external factors such as SES and academic achievement could impact PAS-induced motor facilitation.

Stimulator intensities for RMT and mean SI_1mV_ were high in our study sample. In contrast to Stefan et al. (13) who used between 40 and 50% of maximal stimulator intensity for RMT and mean SI_1mV_, adolescents in our study required 57.4 ± 10.7 and 71.4 ± 11.9% (mean ± SD), respectively. These higher stimulation intensities are similar to those used in previous pediatric TMS studies. Croarkin et al. (41) found intensities of 60.90 ± 5.89% (least squares mean ± SE) and 54.96 ± 6.28% of maximal stimulator output for RMT and SI_1mV_ in depressed adolescents. Similarly, Damji et al. (28) found an RMT of 58.9 ± 14.5% (range 32–92%; mean ± SD) with a mean SI_1mV_ of 68.9 ± 14.3% in non-responders to 63.9 ± 12.2% in definite responders. Our findings further confirm this developmental progression of RMT, supporting findings of Bender et al. (42) who showed RMT and age are inversely related.

Our results suggest PAS can index LTP-like plasticity in healthy adolescents. PAS may, therefore, be a new way with which to study aberrant neuroplasticity in adolescents with mental illness. Research suggests PAS outcomes differ among healthy adults and those with mental illness (10, 43). Since most neuropsychiatric disorders arise in adolescence (8, 44), PAS may allow for identification of mental illness earlier in development, permitting earlier intervention.

Our results also highlight the safety and feasibility of PAS in an adolescent sample. No participants experienced adverse events related to stimulation. Garvey et al. (22) evaluated children’s experiences of single-pulse TMS. Children found TMS more enjoyable than receiving an injection (92%), going to the dentist (84%), throwing up at school (79%), and going on a long car ride (74%). A minority of these children also suggested TMS was more enjoyable than watching television (41%), playing a game (28%), or going to a birthday party (15%). As in Damji et al. (28), all our participants completed the PAS procedure without incident. Together, these findings suggest that PAS could indeed be used to evaluate plasticity in adolescents.

In our study, males, but not females, showed significant PAS-induced CSP lengthening. Since the change in CSP may represent plasticity of GABAergic circuitry, these findings suggest a possible influence of sex hormones on the plasticity of GABA transmission. While direct evidence from human studies is limited, the effect of estradiol on GABA transmission is well characterized in the animal literature. Calza et al. (45) found that neonatal administration of estradiol increased production of α_1_, α_2_, and γ_2_ subunits of cortical GABA_A_ receptors. Similarly, Locci et al. (46) showed that neonatal exposure to estradiol increased the expression of hippocampal extrasynaptic α4/δ subunit-containing GABA_A_ receptors, resulting in improved spatial learning. Carver and Reddy (47) provide a comprehensive review of the literature demonstrating the allosteric effect of neurosteroids on GABA neurotransmission. Another possible explanation for the sex difference found in our sample relates to previous studies that GABA transmission fluctuates with the menstrual cycle. For example, Vigod et al. (48) reported that cortical GABA levels decrease in the mid-follicular phase of the menstrual cycle, during pregnancy, and immediately post-partum. It is possible that plasticity of GABAergic circuitry also decreases during follicular phase, which could partially explain our findings. Alternatively, testosterone may modulate GABAergic interneuron neurocircuitry during development. Animal and human work suggests that sex steroids modulate GABAergic tone (49–51). In male mice, increased testosterone during adolescence accompanies an increase in BDNF (52). BDNF is known to enhance maturation of GABAergic neurons (53). Therefore, the lengthening of CSP in males in our sample could reflect a testosterone-driven effect.

Evidence from animal and human research suggests environmental factors are critical in brain development (54–58). It is unclear why we did not find SES effects in the present study. The mean Hollingshead Index in this sample was 50.6 ± 11.5, reflecting a higher than average SES among participants. Moreover, we tested PAS in the motor cortex. It is possible that SES effects may be more readily revealed in regions, such as the dorsolateral prefrontal cortex (59).

This study had several limitations. The study sample consisted of a majority of females (i.e., 21) with a minority of male participants (i.e., 13). It is possible that this smaller number of adolescent males showed less variability than females. We did not synchronize menstrual cycles or account for menarche in the female participants, potentially introducing variability in the female sample on account of varying brain GABA levels. Still, the variability in male and female CSP duration was comparable across groups as shown in Figure 2. Therefore, we interpret this difference in PAS-induced CSP with caution. In addition, we did not include a sham condition or vary the ISI interval between TMS and PNS pulses as the authors of the original PAS studies had done (13). Future studies could evaluate the impact of sham TMS stimulation, or varying the ISI (between 10 and 100 ms) on PAS-induced motor cortical plasticity.

Our results demonstrate significant PAS-induced motor cortical plasticity in healthy adolescents. Applying PAS to adolescents was safe and well tolerated. We also found significantly greater inhibitory neuroplasticity in young males compared to young females. It is possible that differences in testosterone-induced maturation of GABA neurons or fluctuating GABA levels with female menstrual cycles account for this finding. PAS may eventually serve as an investigational tool in at-risk adolescents, elucidating mechanisms of psychiatric illness.

## Author Contributions

All authors (JL, PC, SA, YS, DB, TR, and ZD) contributed to the conception, structure, and literature review for the manuscript. JL prepared the initial draft, and ZD and PC critically revised the draft. All the authors (JL, PC, SA, YS, DB, TR, and ZD) prepared the final draft, approved the final draft for publication, and agreed to assume accountability for the accuracy and composition of the manuscript.

## Conflict of Interest Statement

JL, SA, TR, and YS report no competing interests. In the last 5 years, PC has received research support from Pfizer for an investigator-initiated study and equipment in-kind support from Neuronetics. DB receives research support and in-kind equipment support for an investigator-initiated study from Brainsway Ltd., and he is the site principal investigator for three sponsor-initiated studies for Brainsway Ltd. He also receives in-kind equipment support from Magventure for an investigator-initiated study. He receives medication supplies for an investigator-initiated trial from Invidior. In the last 5 years, ZD received research and equipment in-kind support for an investigator-initiated study through Brainsway Inc. and a travel allowance through Merck. ZD has also received speaker funding through Sepracor Inc., AstraZeneca and served on the advisory board for Hoffmann-La Roche Limited and Merck and received speaker support from Eli Lilly.

## References

[1] Choudhury S Charman T Blakemore S-J Development of the teenage brain. Mind Brain Educ (2008) 2:142–7. 10.1111/j.1751-228X.2008.00045.x

[2] Moreno M Trainor ME. Adolescence extended: implications of new brain research on medicine and policy. Acta Paediatr (2013) 102:226–32. 10.1111/apa.12100 23176160

[3] Chambers RA Taylor JR Potenza MN. Developmental neurocircuitry of motivation in adolescence: a critical period of addiction vulnerability. Am J Psychiatry (2003) 160:1041–52. 10.1176/appi.ajp.160.6.1041 12777258 PMC2919168

[4] Crews F He J Hodge C. Adolescent cortical development: a critical period of vulnerability for addiction. Pharmacol Biochem Behav (2007) 86:189–99. 10.1016/j.pbb.2006.12.001 17222895 PMC11646682

[5] Insel TR. Rethinking schizophrenia. Nature (2010) 468:187–93. 10.1038/nature09552 21068826

[6] Whittle S Lichter R Dennison M Vijayakumar N Schwartz O Byrne ML Structural brain development and depression onset during adolescence: a prospective longitudinal study. Am J Psychiatry (2014) 171:564–71. 10.1176/appi.ajp.2013.13070920 24577365

[7] Sekar A Bialas AR de Rivera H Davis A Hammond TR Kamitaki N Schizophrenia risk from complex variation of complement component 4. Nature (2016) 530:177–83. 10.1038/nature16549 26814963 PMC4752392

[8] Jones PB. Adult mental health disorders and their age at onset. Br J Psychiatry Suppl (2013) 54:s5–10. 10.1192/bjp.bp.112.119164 23288502

[9] Hasan A Brinkmann C Strube W Palm U Malchow B Rothwell JC Investigations of motor-cortex cortical plasticity following facilitatory and inhibitory transcranial theta-burst stimulation in schizophrenia: a proof-of-concept study. J Psychiatr Res (2015) 61:196–204. 10.1016/j.jpsychires.2014.12.006 25555304

[10] Kuhn M Mainberger F Feige B Maier JG Mall V Jung NH State-dependent partial occlusion of cortical LTP-like plasticity in major depression. Neuropsychopharmacology (2016) 41:1521–29. 10.1038/npp.2015.310 26442602 PMC4832013

[11] Player MJ Taylor JL Weickert CS Alonzo A Sachdev P Martin D Neuroplasticity in depressed individuals compared with healthy controls. Neuropsychopharmacology (2013) 38:2101–8. 10.1038/npp.2013.126 23676792 PMC3773676

[12] Hebb DO The Organization of Behavior. New York: Wiley (1949).

[13] Stefan K Kunesch E Cohen LG Benecke R Classen J. Induction of plasticity in the human motor cortex by paired associative stimulation. Brain (2000) 123:572–84. 10.1093/brain/123.3.572 10686179

[14] Player MJ Taylor JL Weickert CS Alonzo A Sachdev PS Martin D Increase in PAS-induced neuroplasticity after a treatment course of transcranial direct current stimulation for depression. J Affect Disord (2014) 167:140–7. 10.1016/j.jad.2014.05.063 24968188

[15] Batsikadze G Paulus W Kuo MF Nitsche MA. Effect of serotonin on paired associative stimulation-induced plasticity in the human motor cortex. Neuropsychopharmacology (2013) 38:2260–7. 10.1038/npp.2013.127 23680943 PMC3773677

[16] Veniero D Ponzo V Koch G. Paired associative stimulation enforces the communication between interconnected areas. J Neurosci (2013) 33:13773–83. 10.1523/JNEUROSCI.1777-13.2013 23966698 PMC6618662

[17] Ganguly K Schinder AF Wong ST Poo M. GABA itself promotes the developmental switch of neuronal GABAergic responses from excitation to inhibition. Cell (2001) 105:521–32. 10.1016/S0092-8674(01)00341-5 11371348

[18] Croarkin PE Nakonezny PA Husain MM Port JD Melton T Kennard BD Evidence for pretreatment LICI deficits among depressed children and adolescents with nonresponse to fluoxetine. Brain Stimul (2014) 7(2):243–51. 10.1016/j.brs.2013.11.006 24360599

[19] Lewis CP Nakonezny PA Ameis SH Vande Voort JL Husain MM Emslie GJ Cortical inhibitory and excitatory correlates of depression severity in children and adolescents. J Affect Disord (2016) 15(190):566–75. 10.1016/j.jad.2015.10.020 26580570 PMC4685002

[20] Sale MV Ridding MC Nordstrom MA. Factors influencing the magnitude and reproducibility of corticomotor excitability changes induced by paired associative stimulation. Exp Brain Res (2007) 181:615–26. 10.1007/s00221-007-0960-x 17487476

[21] Bhandari A Radhu N Farzan F Mulsant BH Rajji TK Daskalakis ZJ A meta-analysis of the effects of aging on motor cortex neurophysiology assessed by transcranial magnetic stimulation. Clin Neurophysiol (2016) 127:2834–45. 10.1016/j.clinph.2016.05.363 27417060 PMC4956500

[22] Garvey MA Kaczynski KJ Becker DA Bartko JJ. Subjective reactions of children to single-pulse transcranial magnetic stimulation. J Child Neurol (2001) 16:891–4. 10.1177/088307380101601205 11785502

[23] Gilbert DL Garvey MA Bansal AS Lipps T Zhang J Wassermann EM. Should transcranial magnetic stimulation research in children be considered minimal risk? Clin Neurophysiol (2004) 115:1730–9. 10.1016/j.clinph.2003.10.037 15261851

[24] Frye RE Rotenberg A Ousley M Pascual-Leone A. Transcranial magnetic stimulation in child neurology: current and future directions. J Child Neurol (2008) 23:79–96. 10.1177/0883073807307972 18056688 PMC2539109

[25] Rossi S Hallett M Rossini PM Pascual-Leone A Safety, ethical considerations, and application guidelines for the use of transcranial magnetic stimulation in clinical practice and research. Clin Neurophysiol (2009) 120:2008–39. 10.1016/j.clinph.2009.08.016 19833552 PMC3260536

[26] Giedd JN. Structural magnetic resonance imaging of the adolescent brain. Ann N Y Acad Sci (2004) 1021:77–85. 10.1196/annals.1308.009 15251877

[27] Giedd J Stockman M Weddle C Liverpool M Alexander-Bloch A Wallace G Anatomic magnetic resonance imaging of the developing child and adolescent brain and effects of genetic variation. Neuropsychol Rev (2010) 20:349–61. 10.1007/s11065-010-9151-9 21069466 PMC3268519

[28] Damji O Keess J Kirton A. Evaluating developmental motor plasticity with paired afferent stimulation. Dev Med Child Neurol (2015) 57:548–55. 10.1111/dmcn.12704 25640772

[29] Garrett JE Wellman CL. Chronic stress effects on dendritic morphology in medial prefrontal cortex: sex differences and estrogen dependence. Neuroscience (2009) 162:195–207. 10.1016/j.neuroscience.2009.04.057 19401219 PMC2720075

[30] Mychasiuk R Gibb R Kolb B. Prenatal stress alters dendritic morphology and synaptic connectivity in the prefrontal cortex and hippocampus of developing offspring. Synapse (2012) 66:308–14. 10.1002/syn.21512 22121047

[31] Tecchio F Zappasodi F Pasqualetti P De Gennaro L Pellicciari MC Ercolani M Age dependence of primary motor cortex plasticity induced by paired associative stimulation. Clin Neurophysiol (2008) 119:675–82. 10.1016/j.clinph.2007.10.023 18178522

[32] Sirin SR Socioeconomic status and academic achievement: a meta-analytic review of research. Rev Educ Res (2005) 75:417–53. 10.3102/00346543075003417

[33] Caro DH Cortina KS Eccles JS Socioeconomic background, education, and labor force outcomes: evidence from a regional US sample. Sociol Educ (2015) 36:934–57. 10.1080/01425692.2013.868784

[34] Oldfield RC. The assessment and analysis of handedness: the Edinburgh inventory. Neuropsychologia (1971) 9(1):97–113. 5146491 10.1016/0028-3932(71)90067-4

[35] Sheehan DV Sheehan KH Shytle RD Janavs J Bannon Y Rogers JE Reliability and validity of the mini international neuropsychiatric interview for children and adolescents (MINI-KID). J Clin Psychiatry (2010) 71:313–26. 10.4088/JCP.09m05305whi 20331933

[36] Wilkinson GS Robertson GJ Wide Range Achievement Test 4th Edition (WRAT-4) Professional Manual. Lutz, FL: Psychological Assessment Resources (2006).

[37] Hollingshead AB Four-Factor Index of Social Status. Working Paper. New Haven, CT: Yale University (1975).

[38] Stefan K Wycislo M Classen J. Modulation of associative human motor cortical plasticity by attention. J Neurophysiol (2004) 92:66–72. 10.1152/jn.00383.2003 14724259

[39] Säisänen L Pirinen E Teitti S Könönen M Julkunen P Määttä S Factors influencing cortical silent period: optimized stimulus location, intensity and muscle contraction. J Neurosci Methods (2008) 169:231–8. 10.1016/j.jneumeth.2007.12.005 18243329

[40] Jung NH Janzarik WG Delvendahl I Münchau A Biscaldi M Mainberger F Impaired induction of long-term potentiation-like plasticity in patients with high-functioning autism and Asperger syndrome. Dev Med Child Neurol (2013) 55:83–9. 10.1111/dmcn.12012 23157428

[41] Croarkin PE Nakonezny PA Husain MM Melton T Buyukdura JS Kennard BD Evidence for increased glutamatergic cortical facilitation in children and adolescents with major depressive disorder. JAMA Psychiatry (2013) 70:291–9. 10.1001/2013.jamapsychiatry.24 23303429

[42] Bender S Basseler K Sebastian I Resch F Kammer T Oelkers-Ax R Electroencephalographic response to transcranial magnetic stimulation in children: evidence for giant inhibitory potentials. Ann Neurol (2005) 58:58–67. 10.1002/ana.20521 15984026

[43] Frantseva MV Fitzgerald PB Chen R Möller B Daigle M Daskalakis ZJ. Evidence for impaired long-term potentiation in schizophrenia and its relationship to motor skill learning. Cereb Cortex (2008) 18:990–6. 10.1093/cercor/bhm151 17855721

[44] Merikangas KR Nakamura EF Kessler RC. Epidemiology of mental disorders in children and adolescents. Dialogues Clin Neurosci (2009) 11:7–20. 19432384 10.31887/DCNS.2009.11.1/krmerikangas PMC2807642

[45] Calza A Sogliano C Santoru F Marra C Angioni MM Mostallino MC Neonatal exposure to estradiol in rats influences neuroactive steroid concentrations, GABAA receptor expression, and behavioral sensitivity to anxiolytic drugs. J Neurochem (2010) 113:1285–95. 10.1111/j.1471-4159.2010.06696.x 20345753

[46] Locci A Porcu P Talani G Santoru F Berretti R Giunti E Neonatal estradiol exposure to female rats changes GABAA receptor expression and function, and spatial learning during adulthood. Horm Behav (2017) 87:35–46. 10.1016/j.yhbeh.2016.10.005 27769760

[47] Carver CM Reddy DS Neurosteroid interactions with synaptic and extrasynaptic GABAA receptors: regulation of subunit plasticity, phasic and tonic inhibition, and neuronal network excitability. Psychopharmacology (Berl) (2013) 230:151–88. 10.1007/s00213-013-3324-1 24071826 PMC3832254

[48] Vigod SN Strasburg K Daskalakis ZJ Blumberger DM. Systematic review of gamma-aminobutyric-acid inhibitory deficits across the reproductive life cycle. Arch Womens Ment Health (2014) 17:87–95. 10.1007/s00737-013-0403-6 24420415

[49] Jorge JC McIntyre KL Henderson LP. The function and the expression of forebrain GABA(A) receptors change with hormonal state in the adult mouse. J Neurobiol (2002) 50:137–49. 10.1002/neu.10021 11793360

[50] Epperson CN O’Malley S Czarkowski KA Gueorguieva R Jatlow P Sanacora G Sex, GABA, and nicotine: the impact of smoking on cortical GABA levels across the menstrual cycle as measured with proton magnetic resonance spectroscopy. Biol Psychiatry (2005) 57:44–8. 10.1016/j.biopsych.2004.09.021 15607299 PMC4097033

[51] Henderson LP Penatti CA Jones BL Yang P Clark AS. Anabolic androgenic steroids and forebrain GABAergic transmission. Neuroscience (2006) 138:793–9. 10.1016/j.neuroscience.2005.08.039 16310317

[52] Hill RA Wu YW Kwek P van den Buuse M. Modulatory effects of sex steroid hormones on brain-derived neurotrophic factor-tyrosine kinase B expression during adolescent development in C57Bl/6 mice. J Neuroendocrinol (2012) 24:774–88. 10.1111/j.1365-2826.2012.02277.x 22221196

[53] Rasika S Alvarez-Buylla A Nottebohm F. BDNF mediates the effects of testosterone on the survival of new neurons in an adult brain. Neuron (1999) 22:53–62. 10.1016/S0896-6273(00)80678-9 10027289

[54] Duyme M Dumaret A Tomkiewicz S How can we boost IQs of “dull children”? A late adoption study. Proc Natl Acad Sci U S A (1999) 96:8790–4. 10.1073/pnas.96.15.8790 10411954 PMC17595

[55] Bonnier C. Evaluation of early stimulation programs for enhancing brain development. Acta Paediatr (2008) 97:853–8. 10.1111/j.1651-2227.2008.00834.x 18482172

[56] Kolb B Gibb R Brain plasticity and behaviour in the developing brain. J Can Acad Child Adolesc Psychiatry (2011) 20:265–76. 22114608 PMC3222570

[57] Staff RT Murray AD Ahearn TS Mustafa N Fox HC Whalley LJ. Childhood socioeconomic status and adult brain size: childhood socioeconomic status influences adult hippocampal size. Ann Neurol (2012) 71:653–60. 10.1002/ana.22631 22522480

[58] van Praag H Kempermann G Gage FH. Neural consequences of environmental enrichment. Nat Rev Neurosci (2000) 1:191–8. 10.1038/35042057 11257907

[59] Rajji TK Sun Y Zomorrodi-Moghaddam R Farzan F Blumberger DM Mulsant BH PAS-induced potentiation of cortical-evoked activity in the dorsolateral prefrontal cortex. Neuropsychopharmacology (2013) 38:2545–52. 10.1038/npp.2013.161 23820586 PMC3799076

